# The Early Detection of Breast Cancer Using Liquid Biopsies: Model Estimates of the Benefits, Harms, and Costs

**DOI:** 10.3390/cancers14122951

**Published:** 2022-06-15

**Authors:** Esmée K. J. van der Poort, Nicolien T. van Ravesteyn, Jeroen J. van den Broek, Harry J. de Koning

**Affiliations:** 1 Department of Public Health, Erasmus Medical Center, 3015 GD Rotterdam, The Netherlands; jeroen2608@gmail.com (J.J.v.d.B.); h.dekoning@erasmusmc.nl (H.J.d.K.); 2 Department of Biomedical Data Sciences, Leiden University Medical Center, 2333 ZC Leiden, The Netherlands

**Keywords:** liquid biopsy, circulating tumor DNA, breast cancer, screening, cost-effectiveness, ductal carcinoma in situ, digital mammography, overdiagnoses

## Abstract

**Simple Summary:**

Breast cancer screening is associated with benefits, such as mortality reduction and improved quality of life, and harms, such as false-positive results, overdiagnoses, and costs. Novel screen tests could be considered to reduce the harms and increase the benefits of screening. Liquid biopsies have been proposed as a novel method for the early detection of breast cancer. However, studies show that liquid biopsies based on cell-free DNA have a low sensitivity for early-stage breast cancer. Using the microsimulation model MISCAN-Fadia, we model the benefits, harms, and costs of the early detection of breast cancer using liquid biopsies for varying levels of liquid biopsy sensitivity and specificity. We found that liquid biopsies are unlikely to be an alternative to digital mammography, given the test performance based on a CCGA substudy. When liquid biopsies are unable to detect the precursor lesion of breast cancer—ductal carcinoma in situ (DCIS)—they need to be able to detect small, early-stage tumors, with high specificity, at low costs in order to be an alternative to digital mammography. We estimated a maximum liquid biopsy price of USD 187, which is substantially lower than currently listed prices.

**Abstract:**

Breast cancer screening is associated with harms, such as false-positives and overdiagnoses, and, thus, novel screen tests can be considered. Liquid biopsies have been proposed as a novel method for the early detection of cancer, but low cell-free DNA tumor fraction might pose a problem for the use in population screening. Using breast cancer microsimulation model MISCAN-Fadia, we estimated the outcomes of using liquid biopsies in breast cancer screening in women aged 50 to 74 in the United States. For varying combinations of test sensitivity and specificity, we quantify the impact of the use of liquid biopsies on the harms and benefits of screening, and we estimate the maximum liquid biopsy price for cost-effective implementation in breast cancer screening at a cost-effectiveness threshold of USD 50,000. We investigate under what conditions liquid biopsies could be a suitable alternative to digital mammography and compare these conditions to a CCGA substudy. Outcomes were compared to digital mammography screening, and include mortality reduction, overdiagnoses, quality-adjusted life-years (QALYs), and the maximum price of a liquid biopsy for cost-effective implementation. When liquid biopsies are unable to detect DCIS, a large proportion of overdiagnosed cases is prevented but overall breast cancer mortality reduction and quality of life are lower, and costs are higher compared to digital mammography screening. Liquid biopsies prices should be restricted to USD 187 per liquid biopsy depending on test performance. Overall, liquid biopsies that are unable to detect ductal carcinoma in situ (DCIS) need to be able to detect small, early-stage tumors, with high specificity, at low costs in order to be an alternative to digital mammography. Liquid biopsies might be more suitable as an addition to digital mammography than as an alternative.

## 1. Introduction

Breast cancer screening with digital mammography is associated with benefits and harms. Routine screening has been shown to reduce the risk of death by breast cancer up to 40% [1]. However, under the current strategy recommended by the United States Preventive Service Task Force (USPSTF), there are still 953 false-positive screens per 1000 women who are screened biennially from ages 50 to 74 in the U.S., meaning women are likely to receive 1 false-positive result in 13 rounds of screening during their lifetime. In addition, 19 women per 1000 women screened are over-diagnosed with breast cancer [2]. Overdiagnosis is defined as a cancer diagnosis that would otherwise not have caused symptoms or would not have caused death [3]. To overcome the harms of breast cancer screening with digital mammography, novel screening methods should be considered [4].

Liquid biopsies have been proposed as a novel method for the early detection of cancer [5]. Liquid biopsies offer a real-time, minimal-invasive method to detect cancer in the blood through circulating tumor biomarkers [6,7]. Currently, the Circulating Cell-Free Genome Atlas (CCGA) (NCT02889978) study is conducted to develop a blood-based assay based on the sequencing of circulating cell-free DNA (cfDNA) in combination with machine learning to detect multiple cancers early [8]. Two modeling studies show that multi-cancer early detection (MCED) may have a significant impact on population health, especially in cancers without a routine screening program [9,10].

However, due to the low tumor fraction of cfDNA in early-stage cancers [11,12,13,14,15], the suitability of liquid biopsies for early detection is debated [16,17]. There are currently no identified clinical tumor markers for ductal carcinoma in situ (DCIS) [18], the nonobligate precursor lesion of invasive breast cancer. In addition, CCGA and STRIVE (NCT03085888) results for early detection show an average sensitivity of 0% in stage I breast cancer [19] and show mostly detection of tumors with a poor prognosis [20].

In this study, we model the use of liquid biopsies based on cell-free DNA in routine breast cancer screening. We quantify the impact of the use of liquid biopsies with varying combinations of sensitivity and specificity on the harms and benefits of screening, and we estimate the maximum liquid biopsy price for cost-effective implementation in breast cancer screening. In addition, we investigate under what conditions liquid biopsies could be a suitable alternative to digital mammography and compare these conditions to a CCGA substudy [21].

## 2. Materials and Methods

We model the use of liquid biopsies in breast cancer screening in the United States according to three scenarios: (1) liquid biopsies are able to detect ductal carcinoma in situ (DCIS), (2) liquid biopsies are unable to detect DCIS, and (3) liquid biopsies have perfect sensitivity. We compare the harms, benefits, and cost-effectiveness of liquid biopsy screening to screening with digital mammography according to the 2016 U.S. Preventive Service Task Force Recommendation [22].

### 2.1. Model Overview

This study is performed using the breast cancer model of the Erasmus Medical Center, more commonly referred to as MISCAN-Fadia or model E [23], developed within CISNET. The Cancer Intervention and Surveillance Modeling Network (CISNET) aims to estimate the effect of cancer-control interventions on the incidence and risk of death from cancer in the general population [24,25]. The MISCAN-Fadia model, and all other CISNET breast cancer models for that matter, use data from the Surveillance, Epidemiology, and End Results Program and the Breast Cancer Surveillance Consortium [26].

MISCAN-Fadia, short for Microsimulation Screening Analysis-Fatal Diameter, is a discrete event-driven microsimulation model that uses a parallel universe approach. This approach mimics a randomized controlled trial by running scenarios, with and without intervention. The MISCAN-Fadia model has four principal input components: population demographics, screening, the natural history of breast cancer, and treatment. The screening module simulates screening strategies and the proportion of DCIS that is detected by screening, referred to as DCIS sensitivity (%) in this study. The natural history of breast cancer component simulates continuous tumor growth of invasive breast cancer based on several input characteristics, such as tumor biology and age. At tumor onset, tumors have a diameter of 0.01 cm. Each simulated tumor has unique sizes for screen detectability, clinical diagnosis, and fatality. The median size (diameter in cm) of a tumor at screen detectability determines the sensitivity for invasive breast cancer of a screen test. For example, a screen test that detects a tumor at a diameter of 0.01 cm has a 100% sensitivity for invasive BC. The DCIS sensitivity and median tumor size at screen detection together determine in what stage a tumor is detected. The model and the other principal input components have previously been described in more detail [23,27].

For this analysis, we adhered to the current USPSTF strategy where women are screened biennially from ages 50 to 74, with on average 13 screening rounds during their lifetime. We simulated a cohort of 10 million women born in 1970. We counted the outcomes over their lifetime, starting at age 30, assuming that their adherence to screening was 100% and all women diagnosed with breast cancer received treatment.

### 2.2. Model Parameters

#### 2.2.1. Liquid Biopsy Test Characteristics

Currently, digital mammography has a sensitivity of 87% [28], a specificity of 88%, and a cost of USD 149 per mammogram in the U.S. [29]. We ranged sensitivity and specificity to generate several liquid biopsies with combinations of hypothetical test characteristics, summarized in Table 1. We assume liquid biopsies are either able to detect DCIS similar to digital mammography (91% DCIS sensitivity) or not at all (0% DCIS sensitivity). CCGA and STRIVE results show an average sensitivity of 0% in stage I breast cancer [19] and there are currently no identified clinical tumor markers for DCIS [18]. 

We ranged the sensitivity for invasive breast cancer relative to digital mammography by varying the median tumor size for screen detection. MISCAN-Fadia simulates the natural growth of invasive breast cancer tumors and the size at which a tumor is screen-detected according to Weibull distributions. The median tumor size for screen detection refers to the median of the Weibull distribution at which 50% of tumors are screen detectable [27]. 

CCGA and STRIVE report a high specificity for ctDNA liquid biopsies of ~99% [19]. We thus investigated the effect of increasing the specificity from 88% to 96% and 100%. We assumed that liquid biopsy sensitivity and specificity were dependent on age similar to digital mammography. To estimate the impact of a hypothetical perfect test, we investigated the effect of increasing sensitivity for invasive breast cancer to up to 100% (median tumor size of 0.61 and 0.01 cm). In total, we model 46 different combinations of test characteristics.

#### 2.2.2. Health Effects

Using the MISCAN-Fadia model, we estimated the benefits: number of breast cancer deaths and life-years, and harms: false-positives and overdiagnoses. Additionally, we derived disaggregated life-years gained for several components in breast cancer screening, including screening itself, true-positive follow-up, false-positive results, clinical detection, and breast cancer care. Consequently, quality of life adjustments (Table 2) were used to calculate quality-adjusted life-years (QALYs) for each of these components.

#### 2.2.3. Costs

Costs of screening, diagnostics, and treatments were based on published estimates (Table 2) [31,32]. The model produced disaggregated results for the same components as under health effects. We converted all costs to 2020 US dollars.

### 2.3. Analysis

To convert the model inputs to the sensitivities of the modeled liquid biopsies, we estimated the proportion of DCIS and invasive breast cancer that were detected by screening in women aged 50 to 74. This conversion allowed us to compare our model estimates to the results to the CCGA substudy.

We performed the cost-effectiveness analysis using a federal payer perspective, a lifetime horizon, and discounted costs and health effects at 3%, as recommended by the U.S. Public Health Service [33]. Using health utilities for specific health states and costs for specific events, the total costs and QALYs can be derived to calculate cost-effectiveness [27]. The difference in costs divided by the difference in QALYs between a scenario with liquid biopsy and routine screening with digital mammography results in an ICER. The marginal difference in costs between the ICERs and a cost-effectiveness threshold of USD 50,000 was used to calculate the liquid biopsy maximum price, as USD 50,000 is a common cost-effectiveness threshold used in the U.S. [34].

## 3. Results

### 3.1. Proportion of Breast Cancers Detected by Screening

A liquid biopsy with a DCIS sensitivity of 91% and a tumor size of 1.39 cm for screen detection, detected 499,859 cases of invasive breast cancer and 211,799 cases of DCIS in routine screening in women aged between 50 and 74. This resulted in a combined sensitivity for invasive BC and DCIS of 70%. In the scenario where liquid biopsies were unable to detect DCIS, the overall proportion of breast cancers detected by screening was lower. A liquid biopsy with a DCIS sensitivity of 0% and a tumor size of 1.39 cm for screen detection had a combined sensitivity of 62%. In the scenario of a perfect screen test with up to 100% combined sensitivity, considerably more breast cancers were detected by screening and fewer breast cancers were interval detected (Table 3).

### 3.2. Outcomes of Liquid Biopsy Screening

Table 4 presents the modeled liquid biopsies that yielded similar or more QALYs than digital mammography screening and for which a maximum price could be estimated. Overall, our modeling results show that both the benefits and harms of screening in terms of mortality reduction and overdiagnoses increase with a higher sensitivity for invasive breast cancer.

A higher specificity positively influenced QALYs and lowered costs at all levels of sensitivity. In MISCAN-Fadia, specificity does not influence the morality-reduction or the number of overdiagnoses, but does influence the amount of false-positive screens. False-positive screens negatively affect quality-adjusted life years (QALYs) gained and increase the total costs of screening, thereby increasing the eventual liquid biopsy maximum price. Disaggregated costs and QALY decrements per scenario are presented in Table A2 and Table A3 in Appendix A.

**Figure 1:**
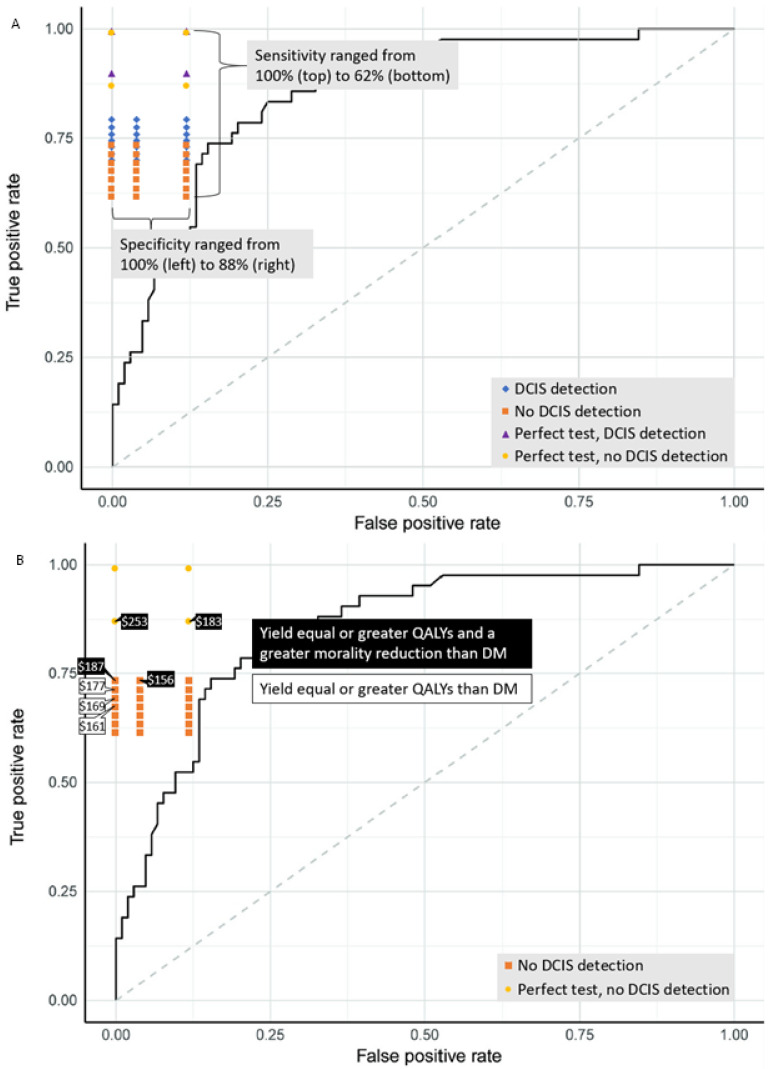
ROC curve of a CCGA breast cancer prediction model adapted from Ref. [21] with the TPR and FPR of the modeled liquid biopsies. (**A**) All modeled liquid biopsies in this study, (**B**) liquid biopsies that are unable to detect DCIS. The costs next to each liquid biopsy represent the maximum price for each liquid biopsy to be cost-effective compared to digital mammography at a threshold of USD 50,000. The filled boxes show the maximum prices where a similar or increased mortality reduction is gained compared to digital mammography screening. Abbreviations: DCIS, ductal carcinoma in situ; DM, digital mammography; TPR, true-positive rate; FPR, false-positive rate.

In the scenario where liquid biopsies are unable to detect DCIS, there were fewer overdiagnoses and a slightly smaller mortality reduction compared to liquid biopsies that are able to detect DCIS. For example, a liquid biopsy that is unable to detect DCIS and has a combined sensitivity of 67%, resulted in a mortality reduction of 23.1%, and 1.9 overdiagnoses per 1000 women compared to a mortality reduction of 25.1% and 18.1 overdiagnoses per 1000 women for a liquid biopsy that is able to detect DCIS. Both liquid biopsies had the same model input for invasive breast cancer. At no DCIS detection, only a combined sensitivity of 73% yielded an increased mortality reduction (26.1%) compared to digital mammography screening. When liquid biopsies are unable to detect DCIS, sensitivity and specificity had to be high for liquid biopsies to be acceptable to digital mammography. We estimated maximum prices between USD 156 and USD 187 for liquid biopsies with a DCIS sensitivity of 0% (Table 4, Figure 1B).

### 3.3. Perfect Screen Test

Additionally, we estimated maximum prices for liquid biopsies approaching 100% sensitivity for invasive breast cancer. At a combined sensitivity of 90% and 87%, the estimated maximum liquid biopsy price ranged from USD 183 to USD 303 depending on the test characteristics. At 99% combined sensitivity, the total costs increased steeply due to the high invasive breast cancer sensitivity. Here, liquid biopsies were less effective and more costly than screening with digital mammography and are therefore not reported in Table 4. (Table A1).

### 3.4. Comparison to a CCGA Substudy

We plotted the true positive rates and false positive rates of the modeled liquid biopsies in our study on the ROC curve of the breast cancer prediction model of a CCGA substudy [21] (Figure 1A) to compare their test performance. The scenario in which liquid biopsies are unable to detect DCIS and have a specificity of 88% seems most concurrent with the predicted results by the CCGA substudy. Our modeled estimates provide threshold values at which a novel screen test would be an acceptable alternative to digital mammography, in terms of the benefits, harms, and cost-effectiveness of screening (Table 4, Figure 1A). The liquid biopsies with a maximum price in Figure 1B yield equal or greater QALYs than digital mammography. The filled labels are the liquid biopsies that also yield a greater mortality reduction than digital mammography screening.

## 4. Discussion

Due to the low tumor fraction of cfDNA in early-stage cancers [11,12,13,14,15] it is questionable whether liquid biopsies can be used in population screening [16]. In this study, we modeled the early detection of breast cancer using liquid biopsies that were either able or unable to detect DCIS, the nonobligate precursor lesion of invasive breast cancer. We estimated the benefits, harms, and costs for different combinations of liquid biopsy sensitivity and specificity. Our findings show that no DCIS detection reduces the number of overdiagnoses by ~90% but also results in a slightly lower mortality reduction. We found that when liquid biopsies are unable to detect DCIS, they need to be able to detect small, early-stage tumors, with high specificity, at low costs in order to be an alternative to digital mammography. We estimated a maximum liquid biopsy price of USD 187 for liquid biopsies fulfilling these requirements. When comparing our model estimates to a CCGA substudy, liquid biopsies seem unlikely to replace digital mammography in routine breast cancer screening given their current test performance.

Our modeling results provide threshold test characteristics for novel tests for breast cancer screening and show how test characteristics affect the harms and benefits of screening. In practice, there is generally a trade-off between sensitivity and specificity. We show that higher specificity increased QoL and decreased costs because of the large number of false-positive screens that were prevented compared to digital mammography screening. For breast cancer sensitivity, MISCAN-Fadia distinguishes between sensitivity for DCIS and sensitivity for invasive breast cancer. A majority of overdiagnosed cases by digital mammography consists of DCIS [2], and, therefore, the number of overdiagnoses decreased when liquid biopsies were unable to detect DCIS but at a lower mortality reduction. In the natural history of breast cancer module of MISCAN-Fadia, pre-clinical detectable DCIS may either develop to clinical DCIS with symptoms, progress to invasive breast cancer, or regress, resulting in no breast cancer [27]. Consequently, women who develop preclinical progressive DCIS or DCIS with symptoms are identified in a later stage of breast cancer, resulting in a higher risk of mortality, lower QoL, and higher costs of breast cancer care. Liquid biopsies that are unable to detect do DCIS should thus have a high sensitivity for invasive breast cancer to be an acceptable, cost-effective alternative to digital mammography. Still, the perfect test scenario shows that even though ~100% sensitivity for invasive breast cancer resulted in a mortality reduction of 70%, this was not cost-effective due to the large number of cases overdetected and the consequent quality of life reductions and costs related to breast cancer care. This emphasizes that a high sensitivity and the consequent greater detection of smaller invasive tumors is not always beneficial in breast cancer screening.

One of the benefits of liquid biopsies as a screen test is the possibility for multi cancer early detection (MCED), especially for cancers with no current screening program [9,10]. However, the use of liquid biopsies in existing screening programs raises a number of challenges. First of all, imaging will always be required after a positive liquid biopsy result for accurate diagnosis and treatment. We take this into account in our study in the diagnostic work-up. In the hypothetical perfect test scenario, liquid biopsies could detect very small tumors that may not yet be detectable by imaging. It will be challenging how women in this instance should be counseled and followed up, e.g., by offering them intense active surveillance and additional imaging until a tumor becomes visible. Second of all, liquid biopsies may not be acceptable as a replacement of the current screen test because of insufficient performance and/or cost-effectiveness. The exact costs of liquid biopsies in a routine screening setting are unclear. In our analysis, we estimate the maximum price for liquid biopsy implementation at a cost-effectiveness threshold of USD 50,000 to be USD 149 to USD 187 per liquid biopsy depending on test performance. Liquid biopsies with comprehensive gene panels, which would be required for population screening, cost USD 1500 to USD 1750 [12]. At that price, liquid biopsies are not suitable for routine testing, although liquid biopsy prices are expected to drop quickly. Analytical methods with a higher sensitivity and lower cost should be considered, such as fragment size analysis and low coverage whole genome sequencing of ctDNA [35].

Although liquid biopsies may not be suitable as a sole screen test, they might be used as an alternative or additional screen test. Liquid biopsies may result in a lower pain experience for women and increase adherence, can be used for tumor profiling and the identification of high risk tumors [20,21], and the identification of individuals with a predisposition for breast cancer [36]. In the STRIVE study, blood samples are taken within 28 days of a screening mammogram, offering the possibility to study the performance of liquid biopsies compared to digital mammography in the future.

There are several limitations to this study that should be considered. First of all, there is no data available on liquid biopsy test performance in a screening setting and, therefore, we had to vary test performances relative to current routine screening. As a consequence, our results should be considered as threshold estimates for the future implementation of liquid biopsies or other novel screening methods but no economic evaluation of an actual, available liquid biopsy. Second, to isolate optimal test performance we assumed adherence to screening and treatment to be 100% in our primary analyses, which most likely will not be attainable in reality. Third, our cost-effectiveness analysis adheres to a federal payer perspective indicating that we did not include all costs from a societal perspective, such as patient time costs. Last, we had to make numerous assumptions on liquid biopsy test characteristics, e.g., the effect of age and tumor biology on the efficacy of liquid biopsies, which might prove different in the future.

In this study, we compared our results to the current USPSTF strategy recommending biennial screening between ages 50 and 74. This guideline factors in the effect of age on breast cancer incidence and digital mammography sensitivity [22]. However, other guidelines recommend different strategies. The American Cancer Society recommends annual screening between ages 45 and 54, followed by biennial screening from age 55 onwards [37], and the American College of Radiology recommends annual screening between ages 40 and 74 [38]. We expect that the impact of liquid biopsies versus digital mammography does not vary much by screening strategy, as the major assumed difference between the two screening modalities is no sensitivity for DCIS. Although the magnitude of benefits and harms may vary slightly, we expect the relative impact on overdiagnoses, breast-cancer mortality, and cost-effectiveness to be similar at earlier starting ages and annual screening intervals.

## 5. Conclusions

This study estimates the benefits, harms, and costs of the early detection of breast cancer using liquid biopsies with varying combinations of test sensitivity and specificity.

When liquid biopsies are unable to detect DCIS, a large proportion of overdiagnosed cases is prevented but overall breast cancer mortality reduction and quality of life are lower, and costs are higher compared to digital mammography screening. In case of no DCIS detection, liquid biopsies should meet to the following requirements in order to be an alternative to digital mammography: sensitivity for detecting invasive disease needs to be high, i.e., liquid biopsies need to be able to also detect small, early-stage tumors, with high specificity, and at low costs. Our study provides threshold values for the use of novel screen tests in breast cancer screening and may guide future evaluation of liquid biopsies as an additional test in breast cancer screening.

## Figures and Tables

**Table 1:** Liquid biopsy test characteristics, costs, and utilities in routine screening.

Type of Screen Test	DCIS Sensitivity, %	Median Tumor Size for Screen Detection, cm	Specificity, %	Cost, USD	Utility
Mammography (comparator)	91	1.21	88	149	0.994
Liquid biopsy	91 or 0	0.01, 0.61, 1.03–1.39 (values ranged per 0.06)	88, 96, or 100	TBD	0.994

Median tumor size for screen detection was ranged from 1.03 cm to 1.39, and to simulate up to 100% sensitivity for invasive breast cancer median tumor size was modeled at 0.01 cm and 0.61 cm. Abbreviations: DCIS, ductal carcinoma in situ.

**Table 2:** MISCAN-Fadia breast cancer model inputs. Screening, diagnostic work-up, and care-related costs (USD), and quality of life effects (QoL).

Screening: Cost per Screen Test and Associated Quality of Life Adjustments				
	Cost, USD	QoL [29]		
Digital mammography	149	0.994 (1 wk)		
Liquid biopsy	TBD	0.994 (1 wk)		
Diagnostic workup: cost by age and screening results and quality of life adjustments				
	False-positive		True positive	
Age group, y	Imaging, USD	Tissue biopsy, USD *	Diagnostics, USD	QoL adjustment [29]
50–64	152	1455	2316	0.895 (5 wks)
65–74	152	1463	2329	0.895 (5 wks)
75–100	152	1550	1964	0.895 (5 wks)
Treatment: cost by stage and phase of care				
	Phase of care ** [30]			
	Initial, USD	Continuous, USD	Terminal, USD	Terminal OCD, USD
In situ	14,848	1336	56,995	7879
Local stage	24,240	2306	59,550	6332
Regional stage	41,352	4474	64,516	10,762
Distant stage	55,985	19,212	81,656	20,081
Treatment: quality of life adjustments by stage and phase of care				
	Phase of care [31,32]			
	Initial, QoL	Continuous, QoL	Terminal, QoL	Terminal OCD, QoL
In situ	0.90	0.93	0.49	0.93
Local stage	0.90	0.93	0.49	0.93
Regional stage	0.75	0.78	0.49	0.78
Distant stage	0.60	0.62	0.49	0.62

All costs were inflated to 2020 US dollars. Abbreviations: QoL, quality of life; OCD, other cause of death. * All women with a false-positive result were assumed to have follow-up imaging. In 10.6% of women with a false-positive result, tissue biopsy was performed in addition to imaging; ** The initial phase of care consists of the first 12 months after diagnosis. The terminal phase of care covers the last 12 months of the life of a woman who has breast cancer. When a woman died of another cause of death (OCD) but had a breast cancer diagnosis during her lifetime, QoL adjustments and costs were applied. The continuous phase of care constitutes the time between the initial and terminal phase.

**Table 3:** Proportion of breast cancers detected by screening.

Model Input		Number of Cancers Detected *				Proportion of Cancers Detected by Screening		
DCIS Sensitivity, %	Median Tumor Size for Screen Detection, cm	Invasive Breast Cancers Detected by Screening	Invasive Breast Cancers Clinically/Interval-Detected	DCIS Detected by Screenings	DCIS Clinically/Interval-Detected	DCIS	Invasive Breast Cancer	Combined Sensitivity
DCIS detection								
91	1.39	499,859	280,403	211,799	25,736	89%	64%	70%
91	1.33	517,279	266,930	211,799	25,701	89%	66%	71%
91	1.27	536,983	251,533	211,799	25,659	89%	68%	73%
91	1.21	555,844	237,439	211,799	25,614	89%	70%	74%
91	1.15	575,086	223,370	211,799	25,558	89%	72%	76%
91	1.09	595,205	208,712	211,799	25,502	89%	74%	78%
91	1.03	617,690	192,351	211,799	25,424	89%	76%	79%
No DCIS detection								
0	1.39	517,043	277,623	0	30,728	0%	64%	62%
0	1.33	535,167	277,623	0	30,728	0%	66%	63%
0	1.27	555,629	261,678	0	30,651	0%	68%	66%
0	1.21	575,168	247,101	0	30,604	0%	70%	67%
0	1.15	595,141	232,510	0	30,547	0%	72%	69%
0	1.09	616,020	217,321	0	30,491	0%	74%	71%
0	1.03	639,366	200,383	0	30,413	0%	76%	73%
Perfect test, DCIS detection								
91	0.61	786,076	87,664	211,799	24,737	90%	90%	90%
91	0.01	1,571,972	2406	211,799	6993	97%	100%	99%
Perfect test, no DCIS detection								
0	0.61	814,739	91,707	0	29,718	0%	90%	87%
0	0.01	1,641,924	2629	0	11,226	0%	100%	99%

* Number of cancers detected between ages 50 and 74 in the entire population of women screened. Abbreviations: DCIS, ductal carcinoma in situ.

**Table 4:** Outcomes for routine screening with liquid biopsy.

Model Inputs		Outcomes per 1000 Women Screened over Their Lifetime					
Combined Sensitivity ^a^, %	Specificity, %	Mortality Reduction %	False Positives	Overdiagnoses	QALYs-Gained	Total Costs, USD 1000	Maximum Price, USD
Digital mammography (comparator)							
74	88	25.1	913.8	18.1	40	7022	-
DCIS detection							
70	100	22.4	0	17.7	43	6786	195
71	96	23.2	406.3	17.8	41	6888	171
71	100	23.2	0	17.8	44	6796	202
73	96	24.2	406.2	18.0	42	6899	179
73	100	24.2	0	18.0	45	6807	210
74	96	25.1	406.1	18.1	44	6911	186
74	100	25.1	0	18.1	47	6819	217
76	88	26.0	913.6	18.2	42	7035	156
76	96	26.0	406.0	18.2	45	6924	193
76	100	26.0	0	18.2	48	6832	224
78	88	27.1	913.4	18.4	43	7050	164
78	96	27.1	405.9	18.4	47	6939	201
78	100	27.1	0	18.4	50	6847	232
79	88	28.3	913.2	18.5	45	7068	173
79	96	28.3	405.8	18.5	49	6957	210
79	100	28.3	0	18.5	52	6868	241
No DCIS detection							
67	100	23.1	0	1.9	41	6951	161
69	100	23.7	0	2.1	42	6964	169
71	100	24.8	0	2.2	44	6979	177
73	96	26.1	409.2	2.4	43	7090	156
73	100	26.1	0	2.4	46	6998	187
Perfect test, DCIS detection							
90	88	38.4	910	20.5	59	7273	235
90	100	38.4	0	20.5	65	7071	303
Perfect test, no DCIS detection							
87	88	36.6	918	4.5	53	7413	183
87	100	36.6	0	4.5	60	7210	253

^a^ Defined as the proportion of breast cancers detected by screening in women aged 50 to 74. Results are presented for liquid biopsies that are an acceptable alternative to DM, i.e., yield equal or greater QALYs than DM and are cost-effective at a cost-effectiveness threshold of USD 50,000. Results are presented compared to no screening. QALYs and total costs were discounted with a rate of 3% per year. Abbreviations: QALYs, quality-adjusted life years; DCIS, ductal carcinoma in situ; DM, digital mammography.

## Data Availability

The data presented in this study are available upon request to the corresponding author. The data are not publicly available due to privacy.

## Appendix A

**Table A1:** Outcomes, cost-effectiveness, and threshold costs for routine screening with liquid biopsies per 1000 women screened over their lifetime.

Median Tumor Size for Screen Detection, cm	Specificity, %	False Positives	QALYs-Gained	Total Costs, USD 1000	ICER, USD 1000 per QALY- Gained	Liquid Biopsy Maximum Price, USD
Liquid biopsy screening with 91% DCIS sensitivity						
1.39	88	914.3	36	6990	-	-
1.39	96	406.3	40	6879	-	-
1.39	100	0	43	6786	−91.2	195
1.33	88	914.2	37	6999	-	-
1.33	96	406.3	41	6888	−162.4	171
1.33	100	0	44	6796	−58.6	202
1.27	88	914.0	39	7010	-	-
1.27	96	406.2	42	6899	−52.3	179
1.27	100	0	45	6807	−39.9	210
1.21	88	913.8	40	7022	-	-
1.21	96	406.1	44	6911	−30.4	186
1.21	100	0	47	6819	−30.4	217
1.15	88	913.6	42	7035	9.3	156
1.15	96	406.0	45	6924	−19.3	193
1.15	100	0	48	6832	−23.4	224
1.09	88	913.4	43	7050	9.5	164
1.09	96	405.9	47	6939	−12.6	201
1.09	100	0	50	6847	−18.2	232
1.03	88	913.2	45	7068	9.5	173
1.03	96	405.8	49	6957	−7.8	210
1.03	100	0	52	6868	−13.7	241
Median tumor size results in up to 100% sensitivity for invasive BC						
0.61	88	910	59	7273	13.5	235
0.61	100	0	65	7071	1.9	303
0.01	88	863	43	10,882	-	-
0.01	100	0	49	10,689	-	-
Liquid biopsy screening with 0% DCIS sensitivity						
1.39	88	922.7	30	7122	-	-
1.39	96	409.7	33	7010	-	-
1.39	100	0	36	6918	-	-
1.33	88	922.5	31	7132	-	-
1.33	96	409.7	35	7020	-	-
1.33	100	0	38	6927	-	-
1.27	88	922.3	33	7143	-	-
1.27	96	409.6	36	7031	-	-
1.27	100	0	39	6939	-	-
1.21	88	922.1	34	7155	-	-
1.21	96	409.5	38	7044	-	-
1.21	100	0	41	6951	−115.1	161
1.15	88	921.9	35	7169	-	-
1.15	96	409.4	39	7057	-	-
1.15	100	0	42	6964	−27.7	169
1.09	88	921.8	37	7184	-	-
1.09	96	409.3	41	7072	86.8	-
1.09	100	0	44	6979	−11.7	177
1.03	88	921.4	39	7202	-	-
1.03	96	409.2	43	7090	27.4	156
1.03	100	0	46	6998	−4.4	187
Median tumor size results in up to 100% sensitivity for invasive BC						
0.61	88	918	53	7413	29.6	183
0.61	100	0	60	7210	9.4	253
0.01	88	869	37	11,173	-	-
0.01	100	0	43	10,979	-	-

Results are presented compared to no screening, except the incremental cost-effectiveness ratios for the liquid biopsies, which are compared to routine screening with mammography and diagnostic follow-up with tissue biopsy. Life-years, quality adjusted life-years, and total costs were discounted with a rate of 3% per year. In case of a negative ICER, a liquid biopsy saved costs and yielded quality-adjusted life years compared to digital mammography. Abbreviations: BC, breast cancer; QALYs, quality-adjusted life years; ICER, incremental cost-effectiveness ratio; DCIS, ductal carcinoma in situ; NAC, not acceptable; DOM, dominated.

**Table A2:** Costs of breast cancer screening and care per 1000 women.

Median Tumor Size for Screen Detection, cm	Specificity, %	Screening and Diagnostics				Care		Total, USD 1000
		Screen Test, USD 1000	False-Positive, USD 1000	True-Positive, USD 1000	Clinical, USD 1000	BC, USD 1000	OCD, USD 1000	
Digital mammography screening								
1.21	88	1173	202.8	122.5	110.3	5140	273.1	7022
Liquid biopsy screening with 91% DCIS sensitivity								
1.03	88	1172	202.7	132.5	102.1	5182	276.1	7068
1.03	96	1172	92.0	132.5	102.1	5182	276.1	6957
1.03	100	1172	-	132.5	102.1	5182	276.1	6865
1.09	88	1173	202.8	129.8	105.1	5165	275.0	7050
1.09	96	1173	92.0	129.8	105.1	5165	275.0	6940
1.09	100	1173	-	129.8	105.1	5165	275.0	6847
1.15	88	1173	202.8	125.5	107.8	5152	274.0	7035
1.15	96	1173	92.0	125.5	107.8	5152	274.0	6924
1.15	100	1173	-	125.5	107.8	5152	274.0	6832
1.21	88	1173	202.9	122.5	110.3	5140	273.1	7022
1.21	96	1173	92.1	122.5	110.3	5140	273.1	6911
1.21	100	1173	-	122.5	110.3	5140	273.1	6819
1.27	88	1173	202.9	119.4	112.8	5129	272.3	7010
1.27	96	1173	92.1	119.4	112.8	5129	272.3	6899
1.27	100	1173	-	119.4	112.8	5129	272.3	6807
1.33	88	1174	202.9	116.2	115.6	5119	271.5	6999
1.33	96	1174	92.1	116.2	115.6	5119	271.5	6888
1.33	100	1174	-	116.2	115.6	5119	271.5	6796
1.39	88	1174	203.0	113.4	118.0	5111	270.8	6989
1.39	96	1174	92.1	113.4	118.0	5111	270.8	6879
1.39	100	1174	-	113.4	118.0	5111	270.8	6786
Perfect test								
0.61	88	1169	202.1	160.6	81.6	5373	287.5	7273
0.61	100	1169	-	160.6	81.6	5373	287.5	7071
0.01	88	1111	192.4	313.7	47.4	8793	423.9	10,882
0.01	100	1111	-	313.7	47.4	8793	423.9	10,689
Liquid biopsy screening with 0% DCIS sensitivity								
1.03	88	1182	204.3	101.6	105.6	5323	286.5	7202
1.03	96	1182	92.7	101.6	105.6	5323	286.5	7090
1.03	100	1182	-	101.6	105.6	5323	286.5	6998
1.09	88	1182	204.4	97.7	108.7	5306	285.3	7184
1.09	96	1182	92.7	97.7	108.7	5306	285.3	7072
1.09	100	1182	-	97.7	108.7	5306	285.3	6979
1.15	88	1183	204.4	93.4	111.4	5293	284.3	7169
1.15	96	1183	92.7	93.4	111.4	5293	284.3	7057
1.15	100	1183	-	93.4	111.4	5293	284.3	6964
1.21	88	1183	204.5	90.2	114.1	5280	283.4	7155
1.21	96	1183	92.7	90.2	114.1	5280	283.4	7044
1.21	100	1183	-	90.2	114.1	5280	283.4	6951
1.27	88	1183	204.5	87.1	116.7	5269	282.5	7143
1.27	96	1183	92.7	87.1	116.7	5269	282.5	7031
1.27	100	1183	-	87.1	116.7	5269	282.5	6939
1.33	88	1183	204.5	83.7	119.5	5259	281.6	7132
1.33	96	1183	92.8	83.7	119.5	5259	281.6	7020
1.33	100	1183	-	83.7	119.5	5259	281.6	6927
1.39	88	1183	204.6	80.9	122.0	5250	280.9	7122
1.39	96	1183	92.8	80.9	122.0	5250	280.9	7010
1.39	100	1183	-	80.9	122.0	5250	280.9	6918
Perfect test								
0.61	88	1178	203.7	129.7	84.3	5519	298.3	7413
0.61	100	1178	-	129.7	84.3	5519	298.3	7210
0.01	88	1119	193.6	289.8	48.3	9081	441.5	11,173
0.01	100	1119	-	289.8	48.3	9081	441.5	10,979

Costs were inflated to USD 2020 and discounted with a rate of 3%. Abbreviations: DCIS, ductal carcinoma in situ; spec, specificity; BC, breast cancer; OCD, other cause of death.

**Table A3:** QALY decrement of breast cancer screening and care per 1000 women.

Median Tumor Size for Screen Detection, cm	Specificity, %	Screening and Diagnostics				Care		Total	Total LYs	Total QALYs
		Screen Test	FP	TP	Clinical	BC	OCD			
Digital mammography screening										
1.21	88	0.91	6.68	0.53	0.51	91.12	2.95	103	39,471	39,368
Liquid biopsy screening with 91% DCIS sensitivity										
1.03	88	0.91	6.68	0.58	0.47	92.23	2.97	104	39,477	39.373
1.03	96	0.91	3.03	0.58	0.47	92.23	2.97	100	39,477	39,377
1.03	100	0.91	-	0.58	0.47	92.23	2.97	97	39,477	39,380
1.09	88	0.91	6.68	0.56	0.48	91.79	2.97	103	39,475	39,371
1.09	96	0.91	3.03	0.56	0.48	91.79	2.97	100	39,475	39,375
1.09	100	0.91	-	0.56	0.48	91.79	2.97	97	39,475	39,378
1.15	88	0.91	6.68	0.55	0.50	91.43	2.96	103	39,473	39,370
1.15	96	0.91	3.03	0.55	0.50	91.43	2.96	99	39,473	39,373
1.15	100	0.91	-	0.55	0.50	91.43	2.96	96	39,473	39,377
1.21	88	0.91	6.68	0.53	0.51	91.12	2.95	103	39,471	39,368
1.21	96	0.91	3.03	0.53	0.51	91.12	2.95	99	39,471	39,372
1.21	100	0.91	-	0.53	0.51	91.12	2.95	96	39,471	39,375
1.27	88	0.91	6.68	0.52	0.52	90.83	2.94	102	39,470	39,367
1.27	96	0.91	3.03	0.52	0.52	90.83	2.94	99	39,470	39,371
1.27	100	0.91	-	0.52	0.52	90.83	2.94	96	39,470	39,374
1.33	88	0.91	6.69	0.51	0.53	90.55	2.94	102	39,468	39,366
1.33	96	0.91	3.03	0.51	0.53	90.55	2.94	98	39,468	39,369
1.33	100	0.91	-	0.51	0.53	90.55	2.94	95	39,468	39,372
1.39	88	0.91	6.69	0.49	0.54	90.31	2.93	102	39,466	39,364
1.39	96	0.91	3.03	0.49	0.54	90.31	2.93	98	39,466	39,368
1.39	100	0.91	-	0.49	0.54	90.31	2.93	95	39,466	39,371
Perfect test										
0.61	88	0.91	6.66	0.70	0.38	97.02	3.07	109	39,496	39,387
0.61	100	0.91	-	0.70	0.38	97.02	3.07	102	39,496	39,394
0.01	88	0.86	6.34	1.37	0.22	167.36	4.45	181	39,552	39,371
0.01	100	0.86	-	1.37	0.22	167.36	4.45	174	39,552	39,378
Liquid biopsy screening with 0% DCIS sensitivity										
1.03	88	0.92	6.75	0.44	0.49	94.66	3.08	106	39,348	39,367
1.03	96	0.92	3.05	0.44	0.49	94.66	3.08	103	39,352	39,371
1.03	100	0.92	-	0.44	0.49	94.66	3.08	100	39,355	39,374
1.09	88	0.92	6.73	0.42	0.50	94.21	3.07	106	39,346	39,365
1.09	96	0.92	3.05	0.42	0.50	94.21	3.07	102	39,350	39,369
1.09	100	0.92	-	0.42	0.50	94.21	3.07	99	39,353	39,372
1.15	88	0.92	6.73	0.41	0.51	93.85	3.07	105	39,345	39,364
1.15	96	0.92	3.05	0.41	0.51	93.85	3.07	102	39,348	39,367
1.15	100	0.92	-	0.41	0.51	93.85	3.07	99	39,352	39,370
1.21	88	0.92	6.74	0.39	0.53	93.52	3.06	105	39,343	39,362
1.21	96	0.92	3.05	0.39	0.53	93.52	3.06	101	39,347	39,366
1.21	100	0.92	-	0.39	0.53	93.52	3.06	98	39,350	39,369
1.27	88	0.92	6.74	0.38	0.54	93.23	3.05	105	39,342	39,361
1.27	96	0.92	3.06	0.38	0.54	93.23	3.05	101	39,346	39,365
1.27	100	0.92	-	0.38	0.54	93.23	3.05	98	39,349	39,368
1.33	88	0.92	6.74	0.36	0.55	92.94	3.04	105	39,341	39,359
1.33	96	0.92	3.06	0.36	0.55	92.94	3.04	101	39,344	39,363
1.33	100	0.92	-	0.36	0.55	92.94	3.04	98	39,347	39,366
1.39	88	0.92	6.74	0.35	0.56	92.70	3.04	104	39,339	38,358
1.39	96	0.92	3.06	0.35	0.56	92.70	3.04	101	39,343	38,362
1.39	100	0.92	-	0.35	0.56	92.70	3.04	98	39,346	39,365
Perfect test										
0.61	88	0.91	6.71	0.56	0.39	99.58	3.19	111	39,362	39,382
0.61	100	0.91	-	0.56	0.39	99.58	3.19	105	39,368	39,388
0.01	88	0.87	6.38	1.26	0.22	172.76	4.63	186	39,335	39,365
0.01	100	0.87	-	1.26	0.22	172.76	4.63	180	39,341	39,382

QALY decrement was calculated by multiplying the life years with (1—utility) for each specific health state. QALYs were consequently calculated as the difference between the total life years and total QALY decrement for each scenario. Life years and QALYs were discounted with a rate of 3%. Abbreviations: DCIS, ductal carcinoma in situ; Spec, specificity; FP, false-positives; TP, true positives; BC, breast cancer; OCD, other cause of death; LYs, life years; QALYs, quality-adjusted life-years.
